# Empagliflozin reduces vascular damage and cognitive impairment in a mixed murine model of Alzheimer’s disease and type 2 diabetes

**DOI:** 10.1186/s13195-020-00607-4

**Published:** 2020-04-07

**Authors:** Carmen Hierro-Bujalance, Carmen Infante-Garcia, Angel del Marco, Marta Herrera, Maria Jose Carranza-Naval, Javier Suarez, Pilar Alves-Martinez, Simon Lubian-Lopez, Monica Garcia-Alloza

**Affiliations:** 1 Division of Physiology, School of Medicine, Universidad de Cadiz, Plaza Fragela sn, 4 piso 410, Cadiz, Spain; 2 Instituto de Investigacion e Innovación en Ciencias Biomedicas de la Provincia de Cadiz (INIBICA), Cadiz, Spain; 3 Salus Infirmorum-Universidad de Cadiz, Cadiz, Spain; 4 Division of Paediatrics, Section of Neonatology, Hospital Universitario Puerta del Mar, Cadiz, Spain

**Keywords:** Alzheimer’s disease, Type 2 diabetes, Amyloid-β, Hemorrhage, Microglia, Tau, Cognition, Empagliflozin

## Abstract

**Background:**

Both Alzheimer’s disease (AD) and type 2 diabetes (T2D) share common pathological features including inflammation, insulin signaling alterations, or vascular damage. AD has no successful treatment, and the close relationship between both diseases supports the study of antidiabetic drugs to limit or slow down brain pathology in AD. Empagliflozin (EMP) is a sodium-glucose co-transporter 2 inhibitor, the newest class of antidiabetic agents. EMP controls hyperglycemia and reduces cardiovascular comorbidities and deaths associated to T2D. Therefore, we have analyzed the role of EMP at the central level in a complex mouse model of AD-T2D.

**Methods:**

We have treated AD-T2D mice (APP/PS1xdb/db mice) with EMP 10 mg/kg for 22 weeks. Glucose, insulin, and body weight were monthly assessed. We analyzed learning and memory in the Morris water maze and the new object discrimination test. Postmortem brain assessment was conducted to measure brain atrophy, senile plaques, and amyloid-β levels. Tau phosphorylation, hemorrhage burden, and microglia were also measured in the brain after EMP treatment.

**Results:**

EMP treatment helped to maintain insulin levels in diabetic mice. At the central level, EMP limited cortical thinning and reduced neuronal loss in treated mice. Hemorrhage and microglia burdens were also reduced in EMP-treated mice. Senile plaque burden was lower, and these effects were accompanied by an amelioration of cognitive deficits in APP/PS1xdb/db mice.

**Conclusions:**

Altogether, our data support a feasible role for EMP to reduce brain complications associated to AD and T2D, including classical pathological features and vascular disease, and supporting further assessment of EMP at the central level.

## Background

Aging remains the main risk factor to suffer Alzheimer’s disease (AD). However, metabolic disorders, and type 2 diabetes (T2D) concretely, are also relevant contributors [1]. In this sense, diabetes has been associated with a 1.5- to 2.5-fold greater risk of dementia (for review, see [2]), and the duration of diabetes has been associated with reduced hippocampal volumes [3]. While the ultimate pathways responsible for the increased risk of dementia in T2D patients have not been elucidated, hyperglycemia, vascular alterations, impaired insulin signaling, and affected amyloid clearance have been pointed out as some of the most relevant underlying possibilities. Moreover, T2D per se is associated with cognitive dysfunction that can range from subtle diabetes-associated cognitive decrements to pre-dementia and dementia [4]. On the other hand, AD has no successful treatment, and therapeutic efforts have been directed towards classical neuropathological features that include gamma-secretase modulators, BACE1 inhibitors, immunotherapy, or tau-based therapies [5–7]. However, different limitations have been raised [8] reducing the presently approved treatments to acetylcholinesterase inhibitors and glutamate antagonist memantine [9]. The close relationship between T2D and AD has resulted in studies based on metabolic approaches, such as intranasal insulin administration [10] or antidiabetic agents [11, 12].

Sodium-glucose co-transporter 2 inhibitors (SGLT2i) are the newest class of oral anti-hyperglycemic agents approved for the treatment of diabetes mellitus [13]. SGLT2i lower glucose levels independent of insulin, by limiting tubular glucose reabsorption. Moreover, among SGLT2i, empagliflozin (EMP) has been deeply analyzed in the EMPA-REG OUTCOME trial, and EMP limits renal disease and reduces cardiovascular and total mortality in patients [14, 15]. Following this idea, it has been suggested that EMP should be considered in patients with T2D and all forms of atherosclerotic cardiovascular disease, to reduce mortality and hospitalization for heart failure [14]. Moreover, the mechanisms responsible for the reductions in cardiovascular risk with EMP remain to be fully elucidated, and the protective cardiorenal effects of EMP seem to be largely independent of its glucose-lowering effects [15]. It has been suggested that SGLT2i, as a glucose-lowering therapy, might be associated with an increased risk of stroke [16]. However it seems that this only reaches statistical significance in specific subgroups (patients under 65 years, patients with increased glycosylated hemoglobin, and patients taking insulin treatment). Therefore, it is also argued that the hazard ratio is within the realm of chance [16]. In addition, long-term treatment with EMP to diabetic mice has shown that learning and memory are largely preserved in db/db mice, and cognitive improvement is accompanied by a reduction of oxidative stress markers in the brain, including reductions in superoxide, 8-OHdG or NADPH oxidase subunits, and gp91phox and p67phox levels. Interestingly, EMP also increased BDNF, as a key protein promoting memory and neuron survival [17]. Likewise, EMP may provide neuroprotection in the diabetic mouse brain by limiting aberrant ultrastructural remodeling of the neurovascular unit, including the neuroglia [18]. Studies with rats on a high-fat diet have also reported the positive effect of SGLT2i treatment on the brain. Cognitive abilities are improved in the Morris water maze (MWM), accompanied by reduced oxidative stress, better insulin signaling, and increased synaptic activity in the hippocampus [19]. Also, SGLT2i improve the performance of mice on a high-fat diet in the new object discrimination test, along with increased neurogenesis in the dentate gyrus and synaptophysin in the striatum oriens [20]. However, to our knowledge, no other studies have assessed the role of SGLT2i on central complications associated to AD and T2D, such as amyloid pathology, microglia activation, spontaneous bleeding, or cognitive deficits. Therefore, in this study, we treat a clinically relevant mixed murine model of AD and T2D (APP/PS1xdb/db mouse) [21, 22] with EMP from 4 to 26 weeks of age, when both AD and T2D are fully established in our mouse model, to assess the direct effect of long-term EMP treatment on brain pathology. As it could be expected, EMP significantly helped to control metabolic alterations. By the end of the treatment, brain atrophy and neuronal loss were significantly reduced in diabetic mice, as well as spontaneous bleeding and microglia burden (Iba1^+^), as a feasible marker of brain inflammation. On the other hand, EMP treatment also reduced senile plaque (SP) burden and amyloid-β (Aβ) levels in AD mice. Moreover, behavioral assessment revealed an overall improvement of learning and memory in treated animals, supporting a role for EMP in brain pathology and behavioral consequences associated with AD and T2D.

## Methods

### Animals, treatments, and metabolic assessment

APP/PS1xdb/db mice were generated in our animal facility by crossing APPswe/PS1dE9 (APP/PS1) [23] and db/db [24] mice, as described [21, 22]. Control, APP/PS1, db/db, and APP/PS1xdb/db mice were randomly divided into groups, and EMP (10 mg/kg) [25] was administered in the diet, as previously described [12], from 4 to 26 weeks of age. Due to the limited amount of animals harboring both AD and T2D, males and females were included in the study, as previously done in similar studies [10, 19, 20]. Body weight as well as glucose and insulin levels was measured before treatment and every 4 weeks until sacrifice at 26 weeks. Immediately after sacrifice, the brains were harvested and weighted. The right hemispheres were dissected and snap-frozen at − 80° until use. The left hemispheres were fixed in paraformaldehyde for 2 weeks before 30-μm sections were cut. All experimental procedures were approved by the Animal Care and Use Committee of the University of Cadiz, in accordance with the Guidelines for Care and Use of experimental animals (European Commission Directive 2010/63/UE and Spanish Royal Decree 53/2013).

### Morris water maze

Spatial memory was assessed 2 weeks before the sacrifice as described [26]. The acquisition phase consisted of 4 trials/day for 4 consecutive days with the platform submerged in quadrant 2. The time limit was 60 s/trial, with a 10-min inter-trial interval. Retention phases were preformed 24 and 72 h after completing the acquisition. The platform was removed, and mice were allowed to swim for 60 s. The time required to locate the platform in the acquisition phase, percentage of time spent in quadrant 2 during the retention phase, and swimming speed were analyzed using the Smart software (Panlab, Spain).

### Motor activity and new object discrimination test

Motor activity assessment and new object discrimination (NOD) commenced after completing the MWM test. Motor activity was assessed by measuring the distance traveled by the mice for 30 min in the open field box. The new object discrimination commenced the next day, and episodic memory paradigms “what,” “where,” and “when” were analyzed as previously described [22].

### Cresyl violet staining

Cresyl violet staining was used to unspecifically label neurons and identify brain regions of interest. Sections located 1 mm apart (from 1.5 to − 3.5 mm from the bregma) were selected, dehydrated in 70% ethanol for 15 min, and incubated in cresyl violet as previously described [12]. Images were acquired in an optical Olympus Bx60 microscope with an Olympus DP71 camera. Cell F (Olympus, Hamburg, Germany) and ImageJ software were used to measure cortical and hippocampal sizes.

### NeuN/DAPI staining

Contiguous sections to those used for cresyl violet staining were incubated with anti-NeuN (Chemicon, CA, USA) (1:200) (Invitrogen, Carlsbad, CA, USA) and Alexa Fluor 594 (Molecular Probes, OR, USA) (1:1000) antibodies, followed by DAPI 1 mg/ml (Sigma, Spain) (1:3000) counterstain. SP were stained with thioflavin S (Sigma, OR) (0.01%) in H_2_O/ethanol (1:1) for 10 min. The percentage of NeuN-positive cells (normalized by total cells stained with DAPI) was quantified in the cortex using ImageJ software as described [12].

### Prussian blue staining

Contiguous sections to those used for NeuN/DAPI staining were incubated with Prussian blue iron staining and neutral red counterstaining, as previously described [22], to analyze spontaneous hemorrhages. The ImageJ software was used to quantify hemorrhage burden, density, and individual hemorrhage size in the cortex and hippocampus.

### Microglia immunostaining

Sections were pre-treated in 70% formic acid and incubated with anti-Iba1 (Wako, Osaka, Japan) (1:1000) and anti-Aβ (4G8, Covance, Greenfield, IN, USA) (1:2000) antibodies overnight at 4 °C in 0.5% BSA, followed by incubation with Alexa Fluor 488 and Alexa Fluor 594 (Molecular Probes, OR, USA) (1:1000). Sections were photographed using a Laser Olympus U-RFL-T fluorescent microscope (Olympus, Japan) and MMIcellTools software. Microglia burden in SP-free areas as well as in the close proximity of SP (up to 50 μm) was quantified as previously described [12].

### SP staining

SP burden was analyzed in the cortex and hippocampus after 4G8 immunostaining and thioflavin S staining. Sections were pre-treated in 70% formic acid and incubated with 4G8 (Covance, Greenfield, IN, USA) antibody (1:2000) followed by Alexa Fluor 594 (Molecular Probes, OR, USA) antibody (1:1000). Sections were further incubated in thioflavin S (Sigma, OR, USA) (0.01%) in H_2_O/ethanol 1:1, mounted, and photographed using a Laser Olympus U-RFL-T fluorescent microscope (Olympus, Japan) and MMIcellTools software. The ImageJ software was used for burden analysis.

### Amyloid beta levels

Soluble and insoluble (formic fraction) Aβ40 and Aβ42 levels were measured in the cortex and hippocampus by colorimetric ELISA kits (Wako, Japan) [12]. Absorbance was measured at 450 nm in a spectrophotometer (MQX200R2, BioTek Instruments, Burlington, VT, USA).

### Total tau and phospho-tau levels

Total tau and tau phosphorylation levels were measured in cortical and hippocampal samples [21]. Phospho-tau clone AT8 (1:1000) (Fisher Scientific, MA, USA) and anti-total tau (1:1000) (DAKO, Denmark) antibodies were used. After normalizing to β-actin (1:2.500.000) (Sigma, USA), optical density was semi-quantified using the ImageJ software. Phospho-tau/total tau ratios were represented as the percentage of control values.

### Statistical analysis

Two-way ANOVA was used for metabolic follow-up and MWM studies. One-way ANOVA for independent samples followed by Tuckey *b* or Tamhane tests or Kruskal-Wallis for independent samples followed by Mann-Whitney *U* test with Bonferroni adjustment were used in the rest of the experiments. The SPSS v.24 software was used for all statistical analysis.

## Results

### EMP ameliorates metabolic alterations in db/db and APP/PS1xdb/db mice

**Fig. 1:**
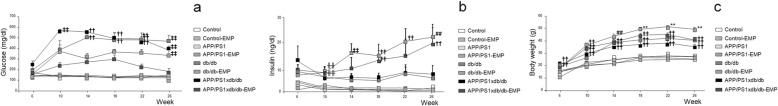
EMP treatment limits metabolic alterations in db/db and APP/PS1xdb/db mice. **a** Long-term EMP treatment significantly reduced postprandial glucose levels in diabetic mice. No differences were detected at week 6 ([*F*_(7, 36)=_0.864, *p* = 0.543], statistical power 0.317), although differences were detected from week 10 to week 22 (week 10 [*F*_(7, 33)_=18.91], week 14 [*F*_(7, 67)_ = 32.92], week 18 [*F*_(7, 69)_ = 31.68], week 22 [*F*_(7, 66)_ = 25.12]; ^‡‡^*p* < 0.01 vs. control, control-EMP, APP/PS1, APP/PS1-EMP, and APP/PS1xdb/db-EMP; ^††^*p* < 0.01 vs. control, control-EMP, APP/PS1, and APP/PS1-EMP]). **b** When insulin levels were analyzed, individual weekly assessment revealed that EMP treatment helped to maintain high insulin levels in db/db and APP/PS1xdb/db mice as the disease progresses (week 6 [*F*_(7, 75)_ = 2.29, *p* = 0.036], week 10 [*F*_(7, 74)_ = 4.47], week 14 [*F*_(7, 69)_ = 5.23], week 18 [*F*_(7, 68)_ = 5.42], week 22 [*F*_(7, 69)_ = 4.49], week 26 [*F*_(7, 69)_ = 6.89]; ^╫╫^*p* < 0.01 vs. control, control-EMP, and APP/PS1]; ^‡‡^*p* < 0.01 vs. control, control-EMP, APP/PS1, APP/PS1-EMP, and db/db; ^††^*p* < 0.01 vs. control, control-EMP, APP/PS1, and APP/PS1-EMP; ^##^*p* < 0.01 vs. control, control-EMP, APP/PS1, APP/PS1-EMP, db/db, and APP/PS1xdb/db). **c** Body weight was maintained by EMP, as revealed by weekly assessment (week 6 [*F*_(7, 75)_ = 6.21], week 10 [*F*_(7, 73)_ = 34.13], week 14 [*F*_(7, 76)_ = 42.39], week 18 [*F*_(7, 77)_ = 44.34], week 22 [*F*_(7, 77)_ = 55.71], week 26 [*F*_(7, 77)_ = 52.55]; ^††^*p* < 0.01 vs. control, control-EMP, APP/PS1, and APP/PS1-EMP]; ^##^*p* < 0.01 vs. control, control-EMP, APP/PS1, APP/PS1-EMP, and db/db; ^††^*p* < 0.01 vs. control, control-EMP, APP/PS1, and APP/PS1xdb/db; **p < 0.01 vs. rest of the groups) (control *n* = 13, control-EMP *n* = 10, APP/PS1 *n* = 9, APP/PS1-EMP *n* = 11, db/db *n* = 11, db/db-EMP *n* = 10–12, APP/PS1xdb/db *n* = 9, APP/PS1xdb/db-EMP *n* = 10)

Postprandial glucose levels were monitored every 4 weeks, from 4 to 26 weeks of age, as detected by 2-way ANOVA (groupXweek) ([*F*_(35, 334)_ = 2.88, **p < 0.01], statistical power 1.000). No differences in glucose levels were present by 6 weeks of age (statistical power 0.317). Severe hyperglycemia was observed in diabetic mice (db/db and APP/PS1xdb/db mice) by 10 weeks of age. While glucose levels were still increased, EMP treatment significantly reduced hyperglycemia in db/db and APP/PS1xdb/db mice, 4 weeks after the commencement of the treatment. Glycemia control was maintained in diabetes-treated mice until the end of the study at 26 weeks of age (Fig. 1a) (statistical power 1.00). No differences were detected by 2-way ANOVA (groupXweek) ([*F*_(35, 424)_ = 1.21, *p* = 0.189], statistical power 0.965) when insulin levels were compared. However, individual weekly assessment revealed that insulin levels were significantly increased in db/db and APP/PS1xdb/db mice along the study. EMP treatment helped to maintain elevated plasmatic insulin in an attempt to control hyperglycemia in diabetic mice, up to 26 weeks of age. These data support a feasible role for EMP to reduce pancreatic exhaustion in db/db and APP/PS1xdb/db mice (statistical power > 0.899) (Fig. 1b). When we analyzed the body weight, we detected a significant groupXweek effect ([*F*_(35, 455)_ = 5.70, **p < 0.01], statistical power 1.000). Body weight was significantly higher in diabetic mice from 6 weeks of age; however, as the disease progresses, the cachectic effect of diabetes is observed. EMP treatment contributed to maintain body weight in db/db and APP/PS1xdb/db mice (statistical power > 1.00) (Fig. 1c), as previously observed with other antidiabetic treatments [21, 22].

### EMP improves learning and memory in AD, T2D, and AD-T2D mice

**Fig. 2:**
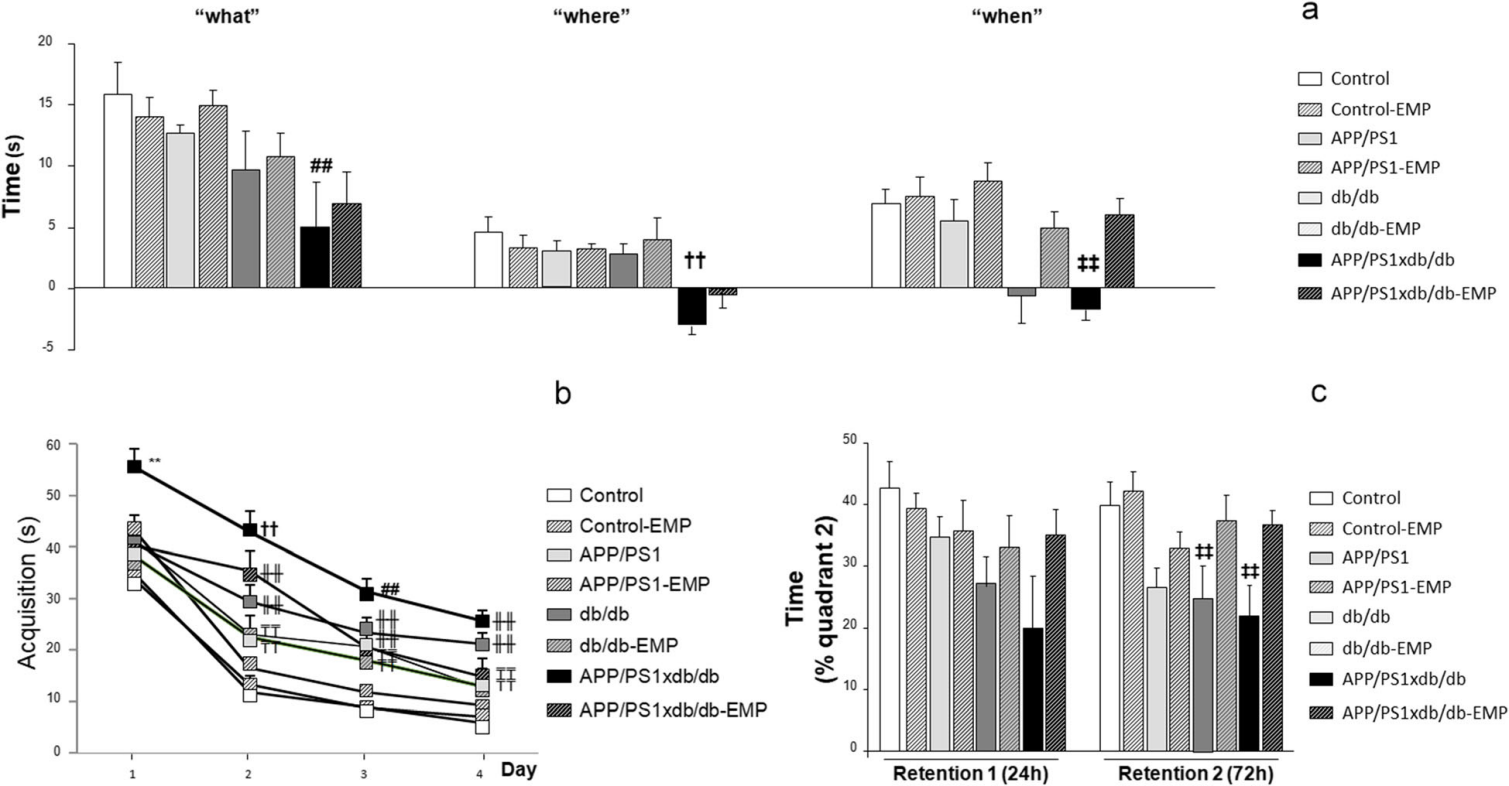
Cognitive impairment in APP/PS1xdb/db mice was ameliorated by EMP treatment. **a** EMP treatment significantly improved the performance in the NOD test for all paradigms under study (“what” [*F*_(7, 207)_ = 2.88], “where” [*F*_(7, 208)_ = 3.17], “when” [*F*_(7, 214)_ = 3.79]; ^##^*p* < 0.01 vs. control, control-EMP, and APP/PS1-EMP; ^††^*p* < 0.01 vs. control, control-EMP, APP/PS1, APP/PS1-EMP, db/db, and db/db-EMP; ^‡‡^*p* < 0.01 vs. control, control-EMP, APP/PS1, APP/PS1-EMP, db/db-EMP, and APP/PS1xdb/db-EMP). **b** In the MWM, EMP treatment reduced acquisition times in APP/PS1, db/db, and APP/PS1xdb/db mice (day 1 [*F*_(7, 298)=_3.85], day 2 [*F*_(7, 288)_ = 14.33], day 3 [*F*_(7, 280)_ = 13.27], day 4 [*F*_(7, 284)_ = 18.24]; **p < 0.001 vs. the rest of the groups; ^††^*p* < 0.001 vs. control, control-EMP, APP/PS1, APP/PS1-EMP, and db/db-EMP; ^╫╫^*p* < 0.01 vs. control, control-EMP, and APP/PS1-EMP; ^₸₸^*p* < 0.01 vs. control and control-EMP). **c** EMP ameliorated the impairment observed along retention 1 (24 h);, however no statistical differences were detected among the groups [*F*_(7, 69)_ = 1.65, *p* = 0.134]. On retention 2 (72 h), reduced cognitive abilities in db/db and APP/PS1xdb/db mice were reversed by EMP treatment [*F*_(7, 68)_ = 4.40, ^‡‡^*p* < 0.001 vs. control and control-EMP] (control *n* = 11, control-EMP *n* = 13, APP/PS1 *n *= 10, APP/PS1-EMP *n* = 10, db/db *n* = 9, db/db-EMP *n* = 10, APP/PS1xdb/db *n* = 5, APP/PS1xdb/db-EMP *n* = 9)

We used a very demanding version of the NOD task, and we observed that episodic memory was slightly affected in APP/PS1 and db/db mice at 26 weeks of age, and the impairment was more severe in APP/PS1xdb/db mice. EMP treatment significantly improved the performance in the NOD test for all paradigms under study (“what,” “where,” and “when,” statistical power > 0.921) (Fig. 2a).

Acquisition in the MWM revealed that APP/PS1 and db/db mice were cognitively impaired, and a synergistic effect was observed in APP/PS1xdb/db mice. EMP treatment reduced acquisition times in APP/PS1, db/db, and APP/PS1xdb/db mice. We detected a significant dayXgroup effect by 2-way ANOVA for independent samples ([*F*_(21, 1150)_ = 1.87, *p = 0.01], statistical power 0.986), and individual day assessment confirmed the improvement as trials were conducted (statistical power > 0.980) (Fig. 2b). Along retention 1 (24 h after completing the acquisition phase), the time spent in quadrant 2 (where the platform used to be located) was reduced for APP/PS1, db/db, and APP/PS1xdb/db mice, whereas EMP treatment ameliorated this situation; however, no statistical differences were detected among the groups (statistical power = 0.640) (Fig. 2c). On retention 2 (72 h after completing the acquisition phase), memory was compromised in APP/PS1, db/db, and APP/PS1xdb/db mice, when compared with wild-type animals. Altered cognitive abilities in db/db and APP/PS1xdb/db mice were reversed by EMP treatment (statistical power = 0.987) (Fig. 2c).

When we measured the total distance traveled, to analyzed motor activity, no significant differences were observed (control, 12052.90 ± 1329.56; control-EMP, 12003.58 ± 681.15; APP/PS1, 11289.92 ± 1012.64; APP/PS1-EMP, 10351.01 ± 974.93; db/db, 11859.18 ± 2197.19; db/db-EMP, 9968.56 ± 763.54; APP/PS1xdb/db, 13422.28 ± 2203.02; and APP/PS1xdb/db-EMP, 8268.43 ± 525.504; [*F*_(7, 69)_ = 1.57, *p* = 0.157], statistical power 0.614), supporting that observed differences in cognitive tests were not derived from altered motor activity.

### Brain atrophy is reduced in diabetic mice after EMP treatment

**Fig. 3:**
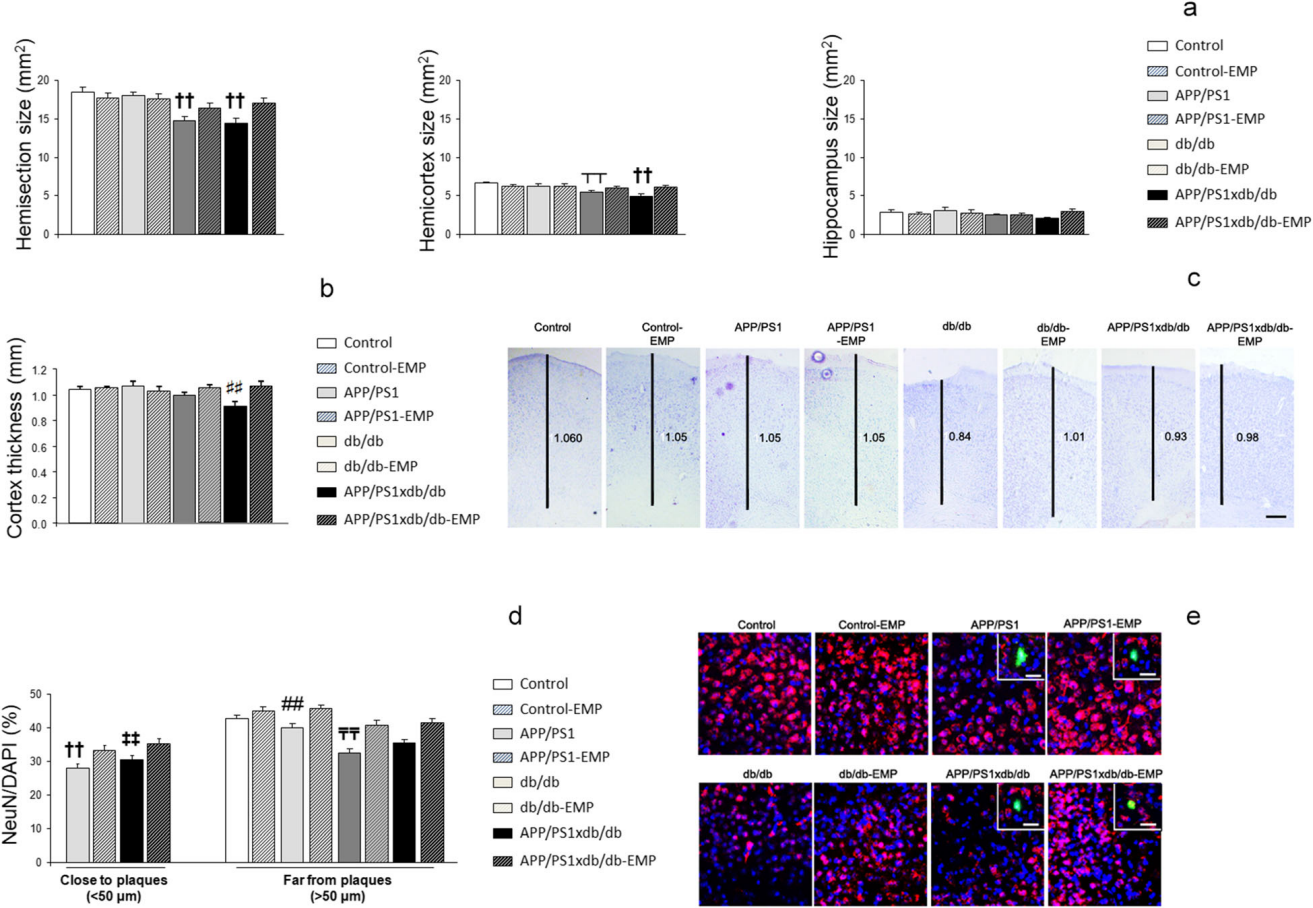
Brain atrophy was ameliorated by EMP treatment. **a** Brain atrophy in db/db and APP/PS1xdb/db mice was improved after EMP treatment, when the hemisection size and cortical areas were analyzed (hemisection [*F*_(7, 205)_ = 6.23]; hemicortex [*F*_(7, 191)_ = 3.59]; ^††^*p* < 0.01 vs. control, control-EMP, APP/PS1, and APP/PS1-EMP]; ^┬^*p* < 0.01 vs. control). While a similar profile was observed in the hippocampus, differences did not reach statistical significance [*F*_(7, 107)_ = 0.718; *p* = 0.657] (control *n* = 7, control-EMP *n* = 9, APP/PS1 *n* = 6, APP/PS1-EMP *n* = 5, db/db *n* = 6, db/db-EMP *n* = 7, APP/PS1xdb/db *n* = 6, APP/PS1xdb/db-EMP *n* = 5). **b** Cortical thickness was also improved after EMP treatment [*F*_(7, 419)=_2.53; ^♯♯^*p* < 0.01 vs. APP/PS, db/db-EMP, and APP/PS1xdb/db-EMP]. **c** Illustrative example of cresyl violet staining where cortical thinning observed in db/db and APP/PS1xdb/db mice is restored by EMP treatment. Scale bar = 125 μm. **d** NeuN/DAPI ratios were reduced in the proximity and far from SP, and EMP treatment reversed this situation (close to plaques [*F*_(3, 933)_ = 6.017, ^††^*p* < 0.001 vs. APP/PS1-EMP and APP/PS1xdb/db-EMP; ^‡‡^*p* < 0.01 vs. APP/PS1xdb/db-EMP]; far from plaques [*F*_(7, 3029)_ = 15.86, ^₸₸^*p* < 0.001 vs. control, control-EMP, APP/PS1, APP/PS1-EMP, db/db-EMP, and APP/PS1xdb/db-EMP; ^##^*p* < 0.01 vs. control-EMP and APP/PS1-EMP]) (control *n* = 5, control-EMP *n* = 4, APP/PS1 *n* = 5, APP/PS1-EMP *n* = 4, db/db *n* = 5, db/db-EMP *n* = 4, APP/PS1xdb/db *n* = 5, APP/PS1xdb/db-EMP *n* = 4). **e** Illustrative example of NeuN/DAPI staining in the proximity and far from SP in the cortex. Scale bars = 25 μm

Brain atrophy in db/db and APP/PS1xdb/db mice was improved after EMP treatment, when the hemisection and cortical areas were measured (statistical power > 0.970) (Fig. 3a, c). While a similar profile was observed in the hippocampus, differences did not reach statistical significance (statistical power 0.276) (Fig. 3a). Similarly, cortical thickness was significantly reduced in diabetic mice, and EMP helped to reverse this situation (statistical power 0.884) (Fig. 3b, c). To further characterize the observed atrophy, we also analyzed the neuronal density in the cortex. NeuN/DAPI ratio was reduced in the cortex from APP/PS1, db/db, and APP/PS1xdb/db mice, both in the proximity of SP and far from plaques, while EMP treatment significantly reversed this situation (statistical power > 0.959) (Fig. 3d, e).

### Spontaneous bleeding is reduced by EMP treatment

**Fig. 4:**
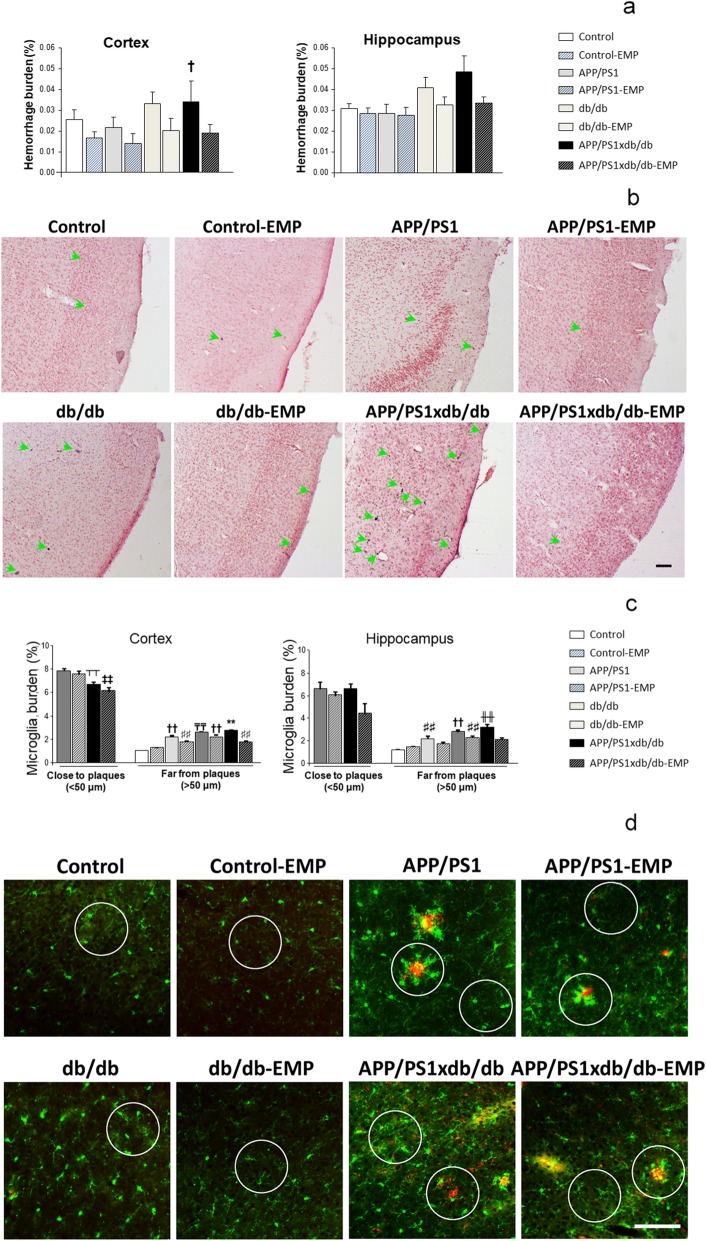
Spontaneous bleeding and inflammation were reduced after EMP treatment. **a** EMP reduced hemorrhage burden in the cortex, and while a similar profile was observed in the hippocampus, differences were not statistically significant (cortex [*F*_(7, 262)_ = 2.65, ^†^*p* = 0.011 vs. control, control-EMP, APP/PS1, and APP/PS1-EMP]; hippocampus [*F*_(7, 118)_ = 01.85, *p* = 0.081]) (control *n* = 8, control-EMP *n* = 6, APP/PS1 *n* = 5, APP/PS1-EMP *n* = 4, db/db *n* = 7, db/db-EMP *n* = 6, APP/PS1xdb/db *n* = 4, APP/PS1xdb/db-EMP *n* = 6). **b** Illustrative example of Prussian blue staining in all groups under study. Green arrows point at individual hemorrhages. Scale bar = 100 μm. **c** Cortical microglia burden was lower in the proximity of SP in APP/PS1xdb/db mice ([*F*_(3, 534)_ = 7.036], ^‡‡^*p* < 0.01 vs. APP/PS1 and APP/PS1-EMP, ^┬┬^*p* < 0.01 vs. APP/PS1]), while in SP-free areas, increased burden was reduced after EMP treatment ([*F*_(7, 4465)=_137.36, **p < 0.01 vs. the rest of the groups, ^₸₸^*p* < 0.01 vs. control, control-EMP, APP/PS1, APP/PS1-EMP, db/db-EMP, and APP/PS1xdb/db-EMP; ^††^*p* < 0.01 vs. control, control-EMP, APP/PS1-EMP, and APP/PS1xdb/db-EMP, ^##^*p* < 0.01 vs. APP/PS1-EMP and APP/PS1xdb/db-EMP]). A similar profile was observed in the hippocampus, and microglia burden was reduced after EMP treatment in SP-free areas (close to plaques ([*F*_(3, 39)_=2.21, *p* = 0.91]); far from plaques [*F*_(7, 768)_ = 23.79, ^╫╫^*p* < 0.01 vs. control, control-EMP, APP/PS1, APP/PS1-EMP, and APP/PS1xdb/db-EMP, ^††^*p* < 0.01 vs. control, control-EMP, APP/PS1-EMP, and APP/PS1xdb/db-EMP, ^♯♯^*p* < 0.01 vs. control and control-EMP]) (control *n* = 5, control-EMP *n* = 5, APP/PS1 *n* = 5, APP/PS1-EMP *n* = 4, db/db *n* = 6, db/db-EMP *n* = 4, APP/PS1xdb/db *n* = 4, APP/PS1xdb/db-EMP *n* = 5). **d** Illustrative example of the cortical areas immunostained for Iba-1 (green) and SP (4G8, red). Circles point out representative regions close and far from SP. Scale bar = 125 μm

As previously described, the burden of spontaneous hemorrhages was higher in db/db and APP/PS1xdb/db mice [21, 22], whereas EMP treatment counterbalanced this situation (statistical power = 0.997) (Fig. 4a, b). This effect was due to a reduction in hemorrhage density, while individual hemorrhage size was not affected (data not shown). We observed a similar profile in the hippocampus, although differences did not reach statistical significance (statistical power = 0.723) (Fig. 4a).

### Iba1^+^ burden is reduced after EMP treatment

Cortical microglia burden was significantly lower in APP/PS1xdb/db mice in the close proximity to SP (statistical power 0.997) in line with previous observations [21, 22]. In SP-free areas, microglia burden was increased in db/db and APP/PS1xdb/db mice and EMP treatment reduced microglia burden (statistical power 1.00) (Fig. 4c, d). While a similar profile was observed in the hippocampus, microglia burden was not significantly affected in areas close to plaques (statistical power = 0.546). In SP-free areas, increased microglia burden in db/db and APP/PS1xdb/db mice was slightly reduced after EMP treatment (statistical power 1.000) (Fig. 4c).

### EMP treatment reduced amyloid pathology in APP/PS1 and APP/PS1xdb/db mice

**Fig. 5:**
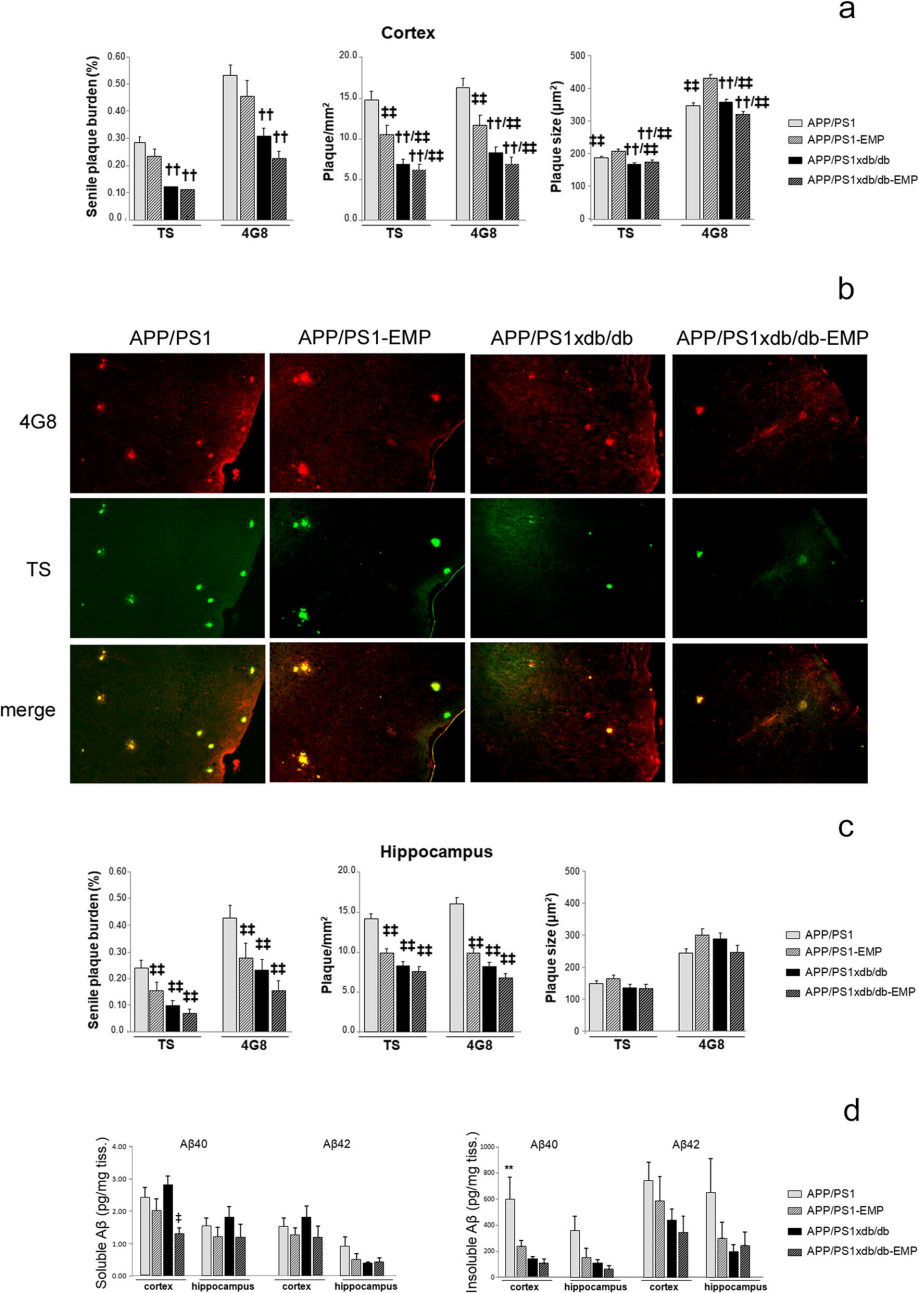
Amyloid pathology is reduced by EMP treatment. **a** SP burden was lower in APP/PS1xdb/db mice when compared with APP/PS1 animals. EMP treatment slightly reduced SP burden in the cortex after TS and 4G8 staining (TS [*F*_(3, 107)_ = 20.55], 4G8 [*F*_(3, 104)_ = 11.89]; ^††^*p* < 0.01 vs. APP/PS1 and APP/PS1-EMP). While individual SP size was increased in APP/PS-EMP mice (TS [*F*_(3, 5191)_ = 8.98], 4G8 [*F*_(3, 5039)_ = 21.11]; ^‡‡^*p* < 0.01 vs. APP/PS1-EMP; ^††^*p* < 0.01 vs. APP/PS1 and APP/PS1-EMP), EMP treatment reduced SP density (TS [*F*_(3, 107)_ = 19.74], 4G8 [*F*_(3, 107)_ = 17.81]; ^‡‡^*p* < 0.01 vs. APP/PS1; ^††^*p* < 0.01 vs. APP/PS1 and APP/PS1-EMP). To a lesser extent, we observed a similar trend when we analyzed SP burden in the hippocampus (TS [*F*_(3, 49)_ = 8.01], 4G8 [*F*_(3, 49)_ = 6.08]; ^‡‡^*p* < 0.01 vs. APP/PS1]), SP density (TS [*F*_(3, 50)_ = 11.57], 4G8 [*F*_(3, 50)_ = 9.42]; ^‡‡^*p* < 0.01 vs. APP/PS1), and individual plaque size (TS [*F*_(3, 643)_ = 0.975, *p* = 0.404]; 4G8 [*F*_(3, 638)_ = 2.39, *p* = 0.067]) (APP/PS1 *n* = 5, APP/PS1-EMP *n* = 5, APP/PS1xdb/db *n* = 5, APP/PS1xdb/db-EMP *n* = 5). **b** Illustrative example of cortical regions stained with TS and 4G8 where reduced SP density can be observed after EMP treatment. Scale bar = 50 μm. **c** A similar profile was observed in the hippocampus when we measured SP burden, density, and individual SP size (^‡‡^*p* < 0.01 vs. APP/PS1) (APP/PS1 *n* = 5, APP/PS1-EMP *n* = 5, APP/PS1xdb/db *n* = 5, APP/PS1xdb/db-EMP *n* = 5). **d** EMP treatment reduced soluble Aβ40 levels in the cortex ([*F*_(3, 27)_ = 4.20, ^‡^*p* = 0.015 vs. APP/PS1xdb/db]). While a similar trend was observed in the hippocampus, differences were not statistically significant ([*F*_(3, 26)_=0.479, *p* = 0.7]). No differences were observed for soluble Aβ42 levels in the cortex ([*F*_(3, 27)_ = 0.832, *p* = 0.488]) or the hippocampus ([*F*_(3, 26)_ = 1.02, *p* = 0.397]). EMP also reduced insoluble Aβ40 levels in the cortex ([*F*_(3, 25)_ = 5.98, **p = 0.003 vs. the rest of the groups]), while differences did not reach statistical significance for insoluble Aβ40 in the hippocampus ([*F*_(3, 22)_ = 2.68, *p* = 0.072]) as well as for insoluble Aβ42 levels (cortex [*F*_(3, 27)_ = 1.43, *p* = 0.254]; hippocampus [*F*_(3, 26)_ = 1.54, *p* = 0.228]) (APP/PS1 *n* = 7, APP/PS1-EMP *n* = 9, APP/PS1xdb/db *n* = 7, APP/PS1xdb/db-EMP *n* = 7)

SP burden was significantly lower in the cortex from APP/PS1xdb/db mice, as previously described in this animal model [21, 22]. EMP treatment slightly reduced SP burden in APP/PS1 and APP/PS1xdb/db mice (statistical power 1.000) (Fig. 5a), although SP were slightly larger in APP/PS1-EMP mice (statistical power > 0.996) (Fig. 5a). However, SP density was reduced by EMP treatment in APP/PS1 animals (statistical power 1.000) (Fig. 5a, b). To a lesser extent, we observed a similar trend when we analyzed SP burden (statistical power > 0.946), density (statistical power > 0.995), and individual plaque size in the hippocampus (statistical power > 0.267) (Fig. 5c).

We also assessed amyloid levels in the cortex and the hippocampus. In line with previous observations, we detected a shift in the natural history of Aβ deposition, and soluble, more toxic species were favored in APP/PS1xdb/db mice, while insoluble Aβ levels were lower, in line with our observations in SP quantification. EMP treatment reduced soluble Aβ40 levels in the cortex (statistical power 0.800) (Fig. 5d). The same profile was observed for Aβ40 levels in the hippocampus (statistical power 0.133), as well as for soluble Aβ42 levels (both in the cortex and hippocampus); however, differences did not reach statistical significance (statistical power > 0.206) (Fig. 5d). EMP also reduced insoluble Aβ40 levels in the cortex (statistical power 0.923) (Fig. 5d), whereas no differences were observed when insoluble Aβ42 levels were compared (statistical power 0.336). Insoluble Aβ40 (statistical power 0.572) and Aβ42 (statistical power 0.357) levels were not significantly affected in the hippocampus (Fig. 5d).

### EMP treatment limits tau phosphorylation

Tau phosphorylation was increased in APP/PS1, and more robustly in db/db and APP/PS1xdb/db mice, as previously described. EMP treatment ameliorated this situation in the cortex from db/db-treated mice (*χ*^2^ = 32.11, *p* = 0.008, ^##^*p* < 0.01 vs. control, statistical power 0.934) (supplementary figure 1a and b). A similar profile was observed in the hippocampus, although differences did not reach statistical significance (*χ*^2^ = 3.74, *p* = 0.809, statistical power 0.337) (supplementary figure 1a and b).

## Discussion

AD has no successful treatment, and patients are in a tremendous need of new therapeutic approaches. The close relationship between T2D and AD has been previously suggested as a relevant target to slow down or reduce brain complications [12, 27, 28]. Following this idea, SGLT2i are the newest oral antidiabetic drugs, and previous studies have focused on characterizing metabolic and peripheral action of these agents. Among SGLT2i, EMP has been shown to control metabolic-related alterations and reduce mortality in patients [29]. However, the role of SGLT2i on the central nervous system has been scarcely addressed in diabetes models [17–19], and to our knowledge, no specific studies have analyzed SGLT2i on AD central pathology.

As expected, EMP ameliorated metabolic alterations, and glucose levels were reduced in diabetic mice. SGLTi mechanism of action is insulin independent; however, since db/db mice are a leptin receptor functional KO model, they are severely diabetic and glucose levels cannot be normalized by EMP treatment. However, we detected that diabetic hyperinsulinemia was longer maintained after EMP treatment, in a similar trend to that previously described in SGLTi-treated db/db mice [30]. These observations are consistent with the maintenance of β-pancreatic activity and insulin levels in diabetic mice after the SGLTi administration [31]. While body weight control has been observed in patients treated with SGLT2i [32, 33], in our hands, long-term EMP treatment maintained body weight, as previously described in db/db cachectic mice treated with SGLTi [30].

Postmortem assessment of EMP-treated mice revealed that brain atrophy observed in diabetic animals was significantly ameliorated. Our previous characterization of db/db [26] and APP/PS1xdb/db mice [21, 22] revealed a preferential affectation of the cortex in these animal models with a slight compromise of the hippocampus. Whereas neuronal loss is not observed in APP/PS1 animals [34], when APP/PS1 mice are crossed with db/db mice, brain atrophy is observed as the disease progresses in APP/PS1xd/db mice, making the model more complex, and better resembling actual AD pathology. In our hands, EMP treatment also helped to keep NeuN/DAPI ratios, suggesting a role for EMP in maintaining the neuroregenerative capacity both in the proximity and far from senile plaques. On the whole, EMP treatment successfully limits cortical thinning and maintains neuronal density after long-term administration, as previously described with other antidiabetic treatments [12, 35, 36].

Vascular disease has been closely associated to AD and T2D, and a synergistic effect has been reported when both pathologies coexist. Spontaneous bleeding is a neuropathological feature that affects db/db mice and more severely APP/PS1xdb/db animals, reproducing brain small vessel disease. In our hands, vascular disease parameters did not correlate with NeuN/DAPI ratios (data not shown), and it is feasible that a combination of all alterations (vascular damage, microglia activation, amyloid pathology, and metabolic alteration) is synergistically responsible for observed cognitive impairment. However, Bello-Chavolla et al. [37] have recently reviewed neuroimaging and neuropathology studies supporting a role for neurodegeneration and cerebrovascular lesions, specifically small vessel disease, in the onset of dementia, consistent with the increased risk of dementia in T2D patients. EMP treatment significantly ameliorated the microhemorrhage burden in the cortex, the most severely affected brain region. These observations are in agreement with previous ultrastructural studies, showing that EMP treatment reduces attenuation and/or loss of pericytes as well as endothelial cell tight and adherents junctions and prevents endothelial dysfunction in different diabetic models [18, 38].

Inflammation is a major component, both in T2D and AD [39], that may underlie the close relationship between T2D and AD [40]. Likewise, hypoglycemic agents show anti-inflammatory activity [41]. Moreover, these anti-inflammatory effects are also detected at the central level with metformin [42], liraglutide [43], multitarget agents [28], or combined therapies [12]. In our hands, EMP also reduced microglia burden in the parenchyma from db/db and APP/PS1xdb/db mice. Our studies are limited to microglia assessment, by Iba1 immunostaining, and therefore, the whole complexity of the inflammatory process is not covered. However, our observations are in accordance with other studies showing that EMP may reduce oxidative stress, astrocyte activation, and inflammation in diabetic models [18, 38]. While the underlying mechanisms have not been completely elucidated, SGLT2i anti-inflammatory activity might be related to increased levels of anti-inflammatory ketone bodies, decreased interface of immune cells with glucose, or reduced insulin and uric acid levels, among others [44].

Amyloid pathology is altered in our AD-T2D model, and more toxic soluble Aβ species are favored in the mixed model; nevertheless, insoluble Aβ is reduced [21, 22]. These observations support a shift in the natural history of Aβ deposition, and whereas soluble Aβ species tend to be increased, insoluble Aβ and SP are reduced. These data are in line with those by Niedowicz et al. [45] reporting an increase of oligomeric Aβ without an effect on Aβ deposition, when AD and T2D are set together, in a similar mouse model. Other studies have reported limited changes in total Aβ levels and increased amyloid angiopathy in young APP23xob/ob mice [46]. Likewise, studies with APP/PS1 mice on a high-fat diet or APP/PS1 mice crossbred with pancreatic insulin-like growth factor 2 overexpressing mice have failed to increase Aβ burden in the brain [47]. We observed that EMP treatment reduces SP density, and an overall reduction of soluble and insoluble Aβ levels is detected in the cortex and the hippocampus from EMP-treated mice. Whereas we cannot point towards a specific mechanism, other antidiabetic treatments have also shown to reduce amyloid pathology [21, 48, 49], based not only on metabolic control but also on their effects on oxidative stress, inflammation, or the blood-brain barrier.

Tau phosphorylation is also increased in diabetic mice, and although EMP reverted this situation in db/db mice, the overall effect was limited. Nevertheless, it also needs to be taken into consideration that APP/PS1 mice do not present overt tau pathology, limiting the outcomes at this level. It seems well established that insulin resistance may increase tau pathology by interfering the balance between tau kinases and phosphatases Tau also modulates insulin signaling [50], contributing to the cross-talk between both pathologies. We have previously observed similar outcomes with other antidiabetic treatments [12]. In line with these observations, pioglitazone has also been shown to reduce tau oligomerization, phospho, and total tau levels, and it also inactivates glycogen synthase kinase 3β in primary neuronal cultures [51].

As previously shown, AD-T2D mice present severe learning and memory compromises [21, 22]. Leptin signaling dysfunction has been associated to central complications, including cognitive impairment [52] or long-term potentiation [53], and therefore, we cannot exclude our findings that it might be at least partially due to altered leptin signaling. However, we observed a better performance of EMP-treated mice in the MWM. Other studies have shown the positive effect of antidiabetic agents in learning and memory [54], and SGLT2i have also been shown to improve cognitive impairment in other metabolic disease models [17, 19].

While to our knowledge, there are no previous studies analyzing the effect of SGLT2i on AD animal models, the beneficial effect of SGLT2i canagliflozin in rats with scopolamine-induced memory impairment has been reported. In this animal model, improvement in the MWM is attributed to the inhibition of acetylcholinesterase activity and monoamine levels in the brain. Canagliflozin might act as a dual acetylcholinesterase and SGLT2i [55], supporting a dual role for SGLT2i at the central level [56, 57] and opening an interesting venue, since acetylcholinesterase inhibitors, and NMDA receptor antagonist memantine, are the only FDA-approved options to treat AD. In our learning and memory studies, we additionally used a very demanding version of the NOD task, which allows the assessment of episodic memory [58]. We also observed an overall improvement after EMP treatment, in line with previous studies [59]. Cognitive enhancement cannot be attributed to a single effect, however it is feasible that by reducing microglia burden, vascular damage, and amyloid pathology, EMP consequently improves learning and memory in AD-T2D mice. Also, the large number of groups in the study also limits the detection of post hoc differences, making it hard to disentangle the direct effect of EMP on AD mice. However, when we analyzed AD mice (APP/PS1 and APP/PS1-EMP) separately, we observed an overall improvement of different pathological features, including cortical NeuN/DAPI ratio, microglia burden in the proximity of SP, or SP density in the cortex and hippocampus, accompanied by better performance in the MWM test, supporting a role for EMP specifically associated to AD pathology in APP/PS1 mice. It also needs to be pointed out that whereas APP/PS1 mice show limited behavioral complications by 6 months of age, db/db mice present severe pathology that limits the chances to take APP/PS1xdb/db mice to older ages. This situation also precludes the possibility to obtain relevant information after the long-term evolution of both pathologies, as commonly observed in the clinic. Nevertheless, APP/PS1xdb/db mice are a relevant model that offers the possibility of assessing both AD and T2D pathological features, offering a more complex version of the process.

## Conclusion

Altogether, our results suggest a role for EMP to reduce brain complications associated to T2D and AD, including classical AD features and vascular disease. Whereas these are initial studies on animal models, the close relationship between AD and T2D may support further assessment of T2D patients treated with EMP, in order to provide new insights into the effects of EMP on the brain.

## Supplementary information

**Additional file 1: Figure 5.** Tau pathology is reduced in db/db mice after EMP treatment.

## Data Availability

Data are available upon reasonable request.

## Abbreviations

AD: Alzheimer’s disease
Aβ: Amyloid-β
EMP: Empagliflozin
MWM: Morris water maze
NOD: New object discrimination
SP: Senile plaque
SGLT2i: Sodium-glucose co-transporter 2 inhibitors
T2D: Type 2 diabetes
