# Supplementary material for: Empagliflozin reduces vascular damage and cognitive impairment in a mixed murine model of Alzheimer’s disease and type 2 diabetes
Source: Alzheimers Res Ther. 2020 Apr 7;12:40. doi: 10.1186/s13195-020-00607-4 (PMC7140573; doi:10.1186/s13195-020-00607-4)

**Empagliflozin reduces VASCULAR DAMAGE AND COGNITIVE IMPAIRMENT in a mixed murine model OF Alzheimer´s disease and Type 2 Diabetes**

Carmen Hierro-Bujalance (1,2), Carmen Infante-Garcia (1,2), Angel del Marco (1,2), Marta Herrera (1), Maria Jose Carranza-Naval (1,2,3), Javier Suarez (1), Pilar Alves-Martinez (1,2,4), Simon Lubian-Lopez (1,2,4), Monica Garcia-Alloza (1,2)

### 1. Division of Physiology. School of Medicine. Universidad de Cadiz.

### 2. Instituto de Investigacion e Innovación en Ciencias Biomedicas de la Provincia de Cadiz (INIBICA).

3. Salus Infirmorum-Universidad de Cadiz.

4. Division of Paediatrics. Section of Neonatology. Hospital Universitario Puerta del Mar. Cadiz.

Address correspondence to:

Monica Garcia-Alloza

Division of Physiology

School of Medicine

Plaza Fragela sn, 4 piso 410

Tel. +34610022298

Email: [monica.garcia@uca.es](mailto:monica.garcia@uca.es)

**Running title**: Empagliflozin reduces brain pathology.

**Figure 5. Tau pathology is reduced in db/db mice after EMP treatment. a)** EMP treatment reduced P-tau levels in the cortex from db/db treated mice (χ^2^=32.11, p=0.008, ##p<0.01 vs. Control). A similar profile was observed in the hippocampus, although differences did not reach statistical significance (χ^2^=3.74 p=0.809) (Control n=7, Control-EMP n=6, APP/PS1 n=7, APP/PS1-EMP=6, db/db n=8, db/db-EMP=6, APP/PS1xdb/db n=5, APP/PS1xdb/db-EMP n=6). **b)** Illustrative western blots with cortical and hippocampal samples from all groups under study.


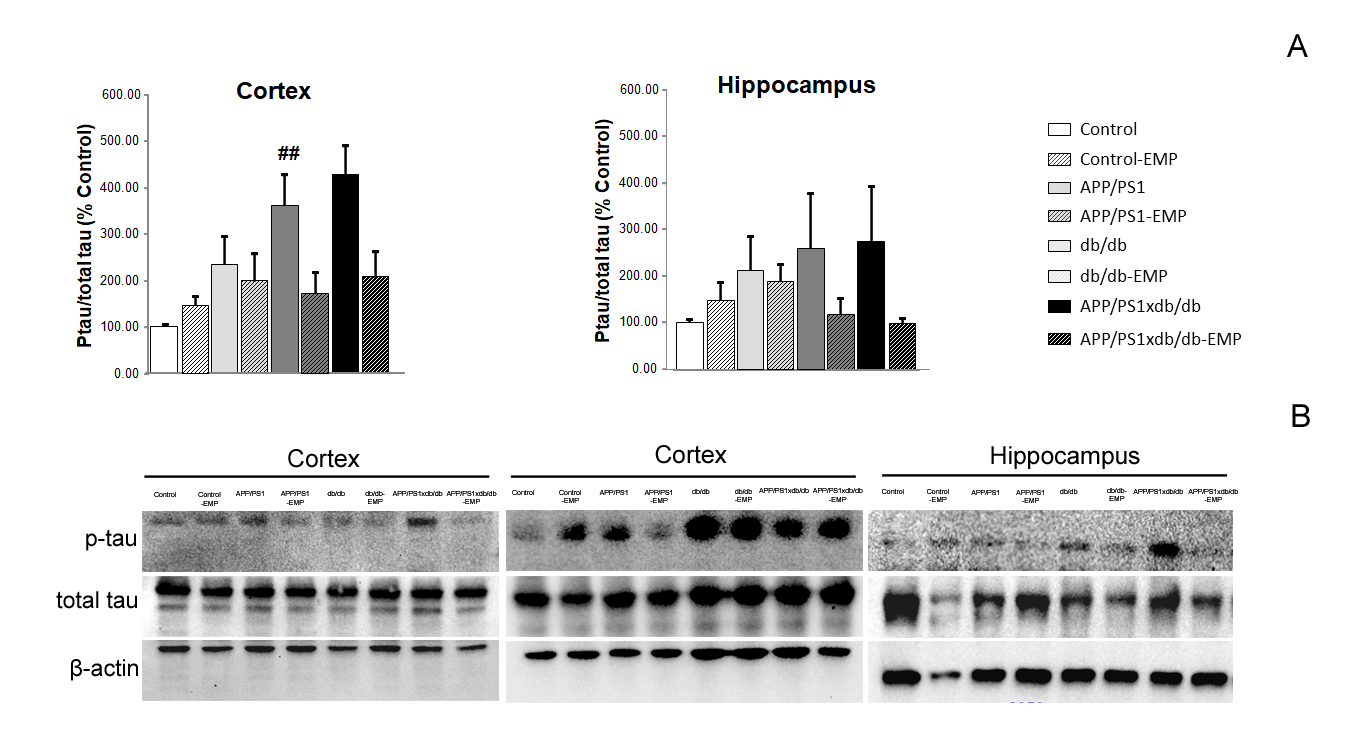

Supplement: Supplementary file 1 — Additional file 1: Figure 5. Tau pathology is reduced in db/db mice after EMP treatment. [file 13195_2020_607_MOESM1_ESM.docx]
